# Working Memory Is Partially Preserved during Sleep

**DOI:** 10.1371/journal.pone.0050997

**Published:** 2012-12-07

**Authors:** Jérôme Daltrozzo, Léa Claude, Barbara Tillmann, Hélène Bastuji, Fabien Perrin

**Affiliations:** 1 CNRS, UMR5292, Lyon Neuroscience Research Center, Auditory Cognition and Psychoacoustics Team, Lyon, France; 2 INSERM, U1028, Lyon Neuroscience Research Center, Auditory Cognition and Psychoacoustics Team, Lyon, France; 3 CNRS, UMR5292, Lyon Neuroscience Research Center, Central Integration of Pain in Human Team, Lyon, France; 4 INSERM, U1028, Lyon Neuroscience Research Center, Central Integration of Pain in Human Team, Lyon, France; 5 Lyon University, Lyon, France; 6 Hospices Civils de Lyon, Sleep Unit, Neurological Hospital, Bron, France; Hangzhou Normal University, China

## Abstract

Although several cognitive processes, including speech processing, have been studied during sleep, working memory (WM) has never been explored up to now. Our study assessed the capacity of WM by testing speech perception when the level of background noise and the sentential semantic length (SSL) (amount of semantic information required to perceive the incongruence of a sentence) were modulated. Speech perception was explored with the N400 component of the event-related potentials recorded to sentence final words (50% semantically congruent with the sentence, 50% semantically incongruent). During sleep stage 2 and paradoxical sleep: (1) without noise, a larger N400 was observed for (short and long SSL) sentences ending with a semantically incongruent word compared to a congruent word (i.e. an N400 effect); (2) with moderate noise, the N400 effect (observed at wake with short and long SSL sentences) was attenuated for long SSL sentences. Our results suggest that WM for linguistic information is partially preserved during sleep with a smaller capacity compared to wake.

## Introduction

Previously, sleep has been considered to be a passive state favoring the recovery of energy. However, recent literature suggests that substantial cerebral activity allowing high cognitive processes occurs during this state. For instance, lexical and sentential semantic processing of auditory speech stimuli have been reported with event-related potentials (ERPs) during “slow wave sleep”, mostly sleep stage 2 (N2), and paradoxical sleep (R) (for a review, see [1]).

According to Baddeley [2], [3], during speech perception, speech-based information is stored and manipulated in working memory (WM). This assumption has been confirmed in language impaired patients and healthy participants [4]–[9]. WM consists of a cognitive system comprising a “central executive system” and a slave system referred to as the “phonological loop”. The active maintenance of internal representations that are necessary to process speech has been shown to depend on the activity of a widely distributed neuronal network including the prefrontal cortex and other brain regions (for a review, see [10]). Up to now, no study (to the knowledge of the author) has explored the preservation/abolition of WM for speech during sleep. Three studies have recorded the N400 component of the ERPs (i.e, a component reflecting semantic incongruency and originating from the prefrontal and temporal areas, see [11], [12]) and found that the meaning of an externally presented semantic material (e.g. word pairs or sentences) was processed during sleep [13]–[15]. Studies using word pairs indicate that lexical processing and storage of a single word in WM could remain during sleep. Based on Ibáñez et al. [14], which tested the perception of sentences, it could be hypothesized that, during N2 and R, sentence-based information (i.e. information that are more complex than lexical-based information) can also be stored and manipulated in WM, even for rather long sentences. Indeed, these authors reported that, during N2 and R, a similar N400 was elicited by the (target) incongruent final word of sentences whether the target was incongruent with the first half of the sentence (“incongruent 1” condition) or the second half of the sentence (“incongruent 2” condition). Even though the main goal of the authors was to test speech perception during sleep and not to optimally control for WM load, it is likely that, for the perception of the semantic incongruence, sentences of the “incongruent 1” condition required more WM capacity (and represented more WM load) than sentences of the “incongruent 2” condition. These results suggest that, during N2 and R, WM may remain efficient for the manipulation of complex and rather extensive speech-based information.

Although still active during N2 and R, WM may nevertheless function differently compared to wake. According to an fMRI study [16], the WM capacity would depend on the activation of the prefrontal and parietal cortices. These authors suggest that, under a high WM load condition, the dorsolateral prefrontal activation would exert a top-down boosting of the WM capacity located in the parietal cortex. Neuroimaging data [17]–[19] have shown that these regions partly overlap regions of hypometabolism during sleep compared to wake, i.e. the dorsolateral prefrontal and the inferior parietal regions. Thus, provided that these regions of hypometabolism are those that control the WM capacity, a reduced capacity of the WM during sleep compared to wake can be expected.

Our present study investigated the extent to which WM for speech processing was preserved during sleep. Unlike Ibáñez et al. [14], we optimally controlled the WM load. Two aspects of WM have been explored. First, we assessed WM capacity using sentences that varied in the amount of speech information required to detect a semantic incongruence. Using a gating paradigm [20], [21], we assessed the amount of words required to perceive the semantic incongruence of the sentences, referred to as the “Sentential Semantic Length” or SSL (see Supplementary Information). We assume that the SSL reflected the load on WM because in a typical WM test assessing the verbal span (e.g. the Speaking Span Test, [22]), a participant is asked to memorize a list of presented words (and then asked to recall these words). The number of words of the list is progressively increased to raise the load on WM. Similarly, during the processing of incongruent sentences, the words of the sentential context corresponding to the SSL had to be stored and manipulated in WM [2], [3] so that a sentential meaning is built from the perception of this sentential context and hence becomes semantically incongruent with the final word.

This allowed us to separate semantically incongruent sentences into two categories: 1) sentences that needed only the last few words of the context to be perceived as incongruent (referred to as “short SSL incongruent sentences”), and 2) sentences that needed more words of the context to be perceived as incongruent (referred to as “long SSL incongruent sentences”; see Methods). Second, we investigated WM capacity by comparing speech perception within a silent or within a noisy background (using two levels of noise yielding to two levels of speech signal degradation). This second experimental manipulation also allowed us to test the WM capacity as the perception of speech-in-noise requires more WM capacity than speech perception without noise [23]–[28]. Indeed, if noise is increased during speech perception, a higher capacity for acoustic-phonetic encoding is required. As stated by Choi et al.[29], “when items […] were presented in noise, perceptual processes required more capacity for acoustic-phonetic encoding, leaving fewer resources for rehearsal […]. This is a specific case of a more general paradigm of presenting two tasks that compete for a general limited capacity […], known as a dual-task paradigm”p1043. More generally, because perceptual processing and rehearsal share overlapping resources in WM, if one process requires more capacity, trade-offs must occur [30].

In sum, our study used the SSL and the level of noise during speech perception to manipulate the load on WM at wake and during sleep. Speech perception was investigated by recording the N400 to sentences. We predicted that, by increasing the load on WM, either with the SSL or with noise, the WM capacity would reach or approach saturation, resulting in less words of the sentential context stored in WM, and hence in an attenuated semantic incongruency of the context with the (target) final word of the sentence. Since the amplitude of the N400 is sensitive to the semantic congruency of a sentential context, the N400 to incongruent sentences was expected to decline (i.e. the difference between the N400 to incongruent and congruent sentences to be reduced) as the load on WM would increase. According to neuroimaging data [16]–[19], we also predicted that WM capacity would be reduced during sleep compared to wake.

## Materials and Methods

### Participants

Sixteen volunteers were tested (13 females, 23.2±0.8 years). All participants were right-handed according to the Edinburgh Handedness Inventory [31], were French native speakers, and did not report any history of neurological disease. All participants had normal hearing, that is, their pure tone auditory thresholds were below 15 dB-HL for frequencies from 250 Hz to 8000 Hz [32]. All participants provided written informed consent to the study, which was conducted in accordance with the guidelines of the Declaration of Helsinki, and approved by the local Ethics Committee (Comité de protection des personnes “Sud-Est II”, 2010-002). One participant did not spend enough time in R to allow the inclusion of the corresponding data in the data analyzes. Therefore, the analyzes were performed only on the data of the remaining 15 participants. Given the difficult context for falling asleep (i.e. the unusual environment of the lab, the video-monitoring, the EEG cap, and the earphones that participants had to wear during sleep), participants were told to awaken two hours earlier to their usual awakening time on the day of the experiment so that they would fall asleep more easily.

### Stimuli

#### Semantic congruency

Fifty sentences ending with a semantically congruent target word were selected from the corpus of an unpublished thesis of phonetics (Monpiou, S. *Approche du rôle du contexte phrastique dans la reconnaissance lexicale auditive des mots*, Doctoral Thesis, Marc Bloch University: Strasbourg, France, 1998) (see Supplementary Information). All these sentences: (a) had a cloze probability (based on the responses of 200 participants) higher than 20 (*M ± SEM = *47.9±3.0) (This means, according to the definition of the cloze probability, that more than 20% of the 200 participants completed spontaneously the missing final word of the sentence by the final word used in our congruent sentences; i.e. the final words of our congruent sentences were highly expected.), (b) had a disyllabic noun target (duration 466 ms to 774 ms, *M ± SEM = *624±13 ms), (c) were presented at a natural speech speed, (d) had a duration of 2 to 3 sec (*M ± SEM = *2.6±0.1), and (e) contained 7 to 13 words (*M ± SEM = *8.8±0.2). The onset of the last word of the sentence (the target, i.e. a disyllabic noun without the article) was estimated from the sentence's acoustic signal using visual (time-frequency signal) and auditory cues (listening to the truncated sentence and the target). Semantically incongruent sentences were built from the same material by reassignments of sentences truncated by the final word (the context) with different targets. One thousand one hundred seventy incongruent sentences were built by reassignment. (The reassignments of 50 truncated sentences with 49 target final words generated 2450 sentences. By removing the syntactically incorrect sentences and the newly created congruent sentences, only 1170 sentences remained.)

The semantic congruency and incongruence of the sentences were tested with a first pilot study (see Supplementary Information).

#### Sentential semantic length (SSL)

A second pilot study was run to estimate the minimum load of sentential context that is required to be stored in WM in order to perceive each of the 1100 incongruent sentences as semantically incongruent (see Supplementary Information). The minimum number of words of the sentential context (i.e. the sentence truncated by its last word) that was required to perceive a sentence as semantically incongruent will be referred to hereafter as “sentential semantic length” (SSL). This number of words counted all types of words, whatever their syntactic function, i.e. including nouns, verbs, pronouns, and articles. An SSL unit may or may not be a unit of meaning or a chunk in WM. Although most of the sentential contextual words were units of meaning, a few other words (e.g. articles or pronouns) may or may not be perceived as units of meaning. Overall, we assume that long SSL incongruent sentences required more units of meaning or chunks stored in WM than short SSL incongruent sentences to be perceived as semantically incongruent.

The implication of the WM load/capacity during the perception of the incongruent sentences was tested with the measured SSL. To this aim, we separated the incongruent sentences into two groups (with short and long SSL) using a median split of the distribution of the SSL of all incongruent sentences (SSL median = 3 words. Short SSL sentences had a context, i.e. sentence truncated by the final target word, of 3 words. Long SSL sentences had a context of 5 words on average, SD = 1.10.). The number of words was 8.77±0.08 for short SSL incongruent sentences and 8.98±0.07 for long SSL incongruent sentences [*t* (1078) = 1.96, *p* = 0.05]. The duration was 498,9±4.2 for short SSL incongruent sentences and 996.1±17.0 for long SSL incongruent sentences [*t* (1078) = 27.4, *p*<.001]. The score of congruency measured in the first pilot study was 1.38±0.03 for short SSL incongruent sentences and 2.05±0.05 for long SSL incongruent sentences [*t* (1078) = 12.3, *p*<.001]. The cloze probability (generally used as an empirical estimate of the contextual constraint, i.e. the degree to which the context establishes an expectation for a particular upcoming word, [12]) was 48.62±0.95 for short SSL incongruent sentences and 48.17±0.85 for long SSL incongruent sentences [*t* (1078) = 0.35, *p* = 0.72].

In this second pilot study, the SSL was measured at wake with sentences being presented without noise or degradation (see next section). The SSL of each sentence at sleep in all degradation conditions (see below) cannot be measured because SSL estimation was based on a behavioral measure. It is likely that the SSL of sentences would be found even smaller (less WM capacity) during sleep according to our prediction (see Introduction). But, most importantly, while the SSL absolute values of each sentence may change according to vigilance state or level of degradation, the SSL rank orders of the sentences are likely to be stable across conditions. Hence, since the dissociation between short and long SSL sentences is based on a rank order statistic, i.e. the median, we assume that the variation of this dissociation across degradation conditions and vigilance states should not affect our comparisons.

#### Degradation

In order to further manipulate the WM load, we tested the perception of speech in silence and in noise (i.e. a more ecologically valid speech perception) with three experimental conditions: sentences presented without noise (Degradation Level 0: DL0), with a “moderate” noise (DL1), and with a “strong” noise (DL3). (A fourth degradation level, called DL2, which was intermediate between DL1 and DL3, was used for another experiment. Here, in order to reduce the amount of experimental conditions in an attempt to keep a good signal to noise ratio, we did not present the sentences under the DL2 condition.) The full sentences (i.e. the context and the target) were acoustically degraded. The strong degradation (DL3) was performed by modulation of the acoustic signal [33] with a pink noise (i.e. a noise that compensates for the ear sensitivity) (signal-to-noise ratio range: −2.85 to 0.08 dB according to Adobe Audition 1.5). The moderate degradation (DL1) was obtained with the same procedure except that before modulation, the pink noise was low-pass filtered using a Fast Fourier Transformation at 4000 Hz (i.e. the highest frequencies of the speech signal above 4000 Hz were not degraded) (signal-to-noise ratio range: −3.24 to −0.30 dB according to Adobe Audition 1.5).

The sound level of all sentences was normalized with the dB-A mean RMS. Sentences were delivered binaurally to the participants with an inter-stimulus interval of 1s. The mean sound level of sentences was delivered at 54.5 dB-A and, during sleep, was then decreased by steps of 3 dB-A to a sound level which did not interrupt the participant sleep. The mean sound level of sentences across participants and vigilance states was 45.7±1.2 dB-A (range: 39.5 to 54.5 dB-A) according to a sound level meter and a standard artificial ear (Larson Davis AEC101 and 824). All stimuli were played binaurally through a soundcard (Creative SB X-Fi Audio) connected to earphones with sound tubes (ER-2 Etymotic).

### Procedure

#### Stimulation Procedure

During the entire experiment, participants listened to 2200 sentences: 50 congruent sentences that were presented 22 times and 1100 incongruent sentences that were not repeated within participants. Half of the incongruent sentences were associated to a short SSL, the other half to a long SSL. One third of all (congruent and incongruent) sentences were presented without noise (DL0), one third with moderate noise (DL1), and one third with a strong noise (DL3). We recorded 2200 trials per participant with the 50 congruent sentences repeated 22 times and 1100 different incongruent sentences, presented in a pseudo-random order (with a new randomization for each participant) and across 3 vigilance states (wake, N2, and R). Approximately 50 trials per experimental condition entered in the statistical analyzes (after EEG artifacts correction, see next section), with 27 experimental conditions (3 types of sentences: congruent, short SSL incongruent, and long SSL incongruent, 3 degradation levels, and 3 vigilance states) (see the number of recorded trials per experimental condition in the Supplementary Information).

We assumed that the N400 component of the ERPs in response to the final word of a sentence is reduced by sentence repetition only when the sentence is ending on a semantically incongruent target, but not on a congruent target [34]. This hypothesis was investigated by comparing the ERPs during wake before and after sleep. Half of the participants were presented with sentences during wake before sleep (group 1) and then during sleep, and the other half of the participants were presented the sentences during sleep and then during wake after sleep (group 2) (with repetition of the congruent sentences between wake and sleep but no repetition of the incongruent sentences between wake and sleep). If a repetition effect on the N400 in response to the congruent sentences occurred, the N400 to the congruent sentences during wake of group 2 would be attenuated compared to group 1, hence the N400 effect would differ between these two groups. However, there was no significant difference between their ERP responses (*p*>.05). As a consequence, the waking data of the two groups were averaged together in the following analyzes. Moreover, we assumed that in the present study, the repetition of the congruent sentences did not influence the ERPs (in terms of a reduction of the N400 for instance). This assumption covered the full latency window of the recorded ERPs on the basis of our comparison between ERPs of group 1 and 2. This is consistent with the literature for the N400 [34]. However, repetition effects of congruent sentences have been reported with late positive components [35]. This discrepancy may be related to the presentation of most of our congruous sentences during sleep and/or under a degradation condition.

The goal of our study was to explore WM during speech perception under several stages of sleep (as defined by the AASM standard, [36]). Given that N1 has a low probability of occurrence during sleep [37] and that stimulation during N3 resulted almost systematically in either awakening or entrance into N1 or N2 (according to the online-monitored polysomnographic pattern), these sleep stages were not explored. In addition to N2 and R, we analyzed participants' responses during wake, as a control condition.

#### Electroencephalography

Electroencephalographic (EEG) signals from 18 Ag-AgCl electrodes (International 10–20 system sites: Fz, Cz, Pz, Oz, F7, F8, F3, F4, C3, C4, T7, T8, P7, P8, P3, P4, O1, O2) referenced to the nose, horizontal electrooculograms (EOG) from bipolar electrodes positioned at the outer canthi of both eyes, vertical EOGs from bipolar electrodes positioned below and above the left eye, and bipolar submental electromyogram were amplified using the Brain Quick SD64 Micromed system and sampled at 512 Hz (16 bits) using an analog bandpass filter of .05–128 Hz. A ground electrode was placed between Fz and FPz site and the impedance at all electrodes was kept below 5 kΩ. All analyzes were performed using custom scripts written in Matlab (The MathWorks) and the EEGLAB toolbox [38]. Signals containing high amplitude K-complex and non-stereotypical artifacts, including high-amplitude, high-frequency muscle noise and electrode cable movements, were rejected (about 25% of the trials). Stereotypical artifacts such as eye movements and eye blinks were corrected with an extended Infomax independent component analysis [39] implemented in EEGLAB. In this analysis, the data were broken into 18 component activations per participant, component activations representing non-brain artifacts were removed (based on a visual inspection of their scalp topographies, time courses, and frequency spectra) and EEG data were reconstructed from the remaining component activations [38].

After the installation of the head cap and of two mini-earphones inserted into the external acoustic canals, participants laid down on a comfortable bed in a sound-attenuated and electromagnetically shielded room with infrared video-monitoring. The EEG, received through an optic cable, was monitored in an adjacent room. Before going to sleep, if the participant belonged to the group where waking (control) data were recorded before sleep (see above), they were instructed to sit on the side of the bed and to stay awake with opened eyes and to listen carefully to the stimuli without any specific task (passive condition) for approximately 40 minutes. The stimuli were presented during three blocks of about 13 mn. If the participant showed signs of falling asleep according to the online-monitored polysomnographic pattern, the stimulation was immediately discontinued and the participant was told again to stay awake and to listen carefully to the stimuli. Then participants were told that stimuli would be presented during sleep (but not the nature of these stimuli, i.e. sentences) and that they could sleep now. The room light was turned off. The experimenter closed the room and moved to the adjacent EEG- and video-monitoring room. During sleep, they were stimulated approximately every 20–30 minutes in both the first and the second parts of the night. If a stimulus awakened the sleeper (according to the online-monitored polysomnographic pattern, with an increased alpha or beta activity, or movements according to the EMG or the video-monitoring, or eyes opening according to the EOG or the video-monitoring), the stimulation sequence was immediately discontinued. Thus, the number of rejected trials due to awakening was extremely low, approximately 10 trials per participants (i.e. less than 0.5% of the presented trials). The experimenter checked regularly that the earphones were kept in the participant's ears (by coming silently next to the sleeper with a small headlight while the room light was kept off), particularly before and after a series of stimulation and when the participant's EMG or video-monitoring indicated movements. After awakening, if the participant belonged to the group where waking (control) data were recorded after sleep (see above), they were instructed to sit on the side of the bed and to stay awake with opened eyes and listen carefully to the stimuli without any specific task (passive condition) for approximately 40 minutes (see wake stimulation above). The present study was performed without adaptation night.

#### Data analyzes

Sleep stages were visually scored off-line by two investigators of the study according to the criteria of the AASM standard [36] (see polysomnographic samples and EEG spectra in the Supplementary Information) so as to derive hypnograms based on 30s epochs and determine the vigilance state during which stimuli were delivered. More restricted criteria were used during the intervals immediately preceding and following each stimulation in order to reject: (a) cortical responses occurring during an arousal period, and (b) responses obtained following less than 1 minute of continuous sleep.

Individual event-related potentials (ERPs), time-locked to the sentence final word's onset, were analyzed over a 1600 ms epoch, including a prestimulus baseline of 200 ms, and were grouped according to the vigilance state (wake vs. N2 without concomitant K-complex vs. R), blind to the type of stimulus. ERPs were then averaged according to the type of sentence (congruent sentence vs. short SSL incongruent sentence vs. long SSL incongruent sentence), the degradation level (DL0 vs. DL1 vs. DL3, see Stimuli section above) and the electrode position. Prior to averaging, single epochs containing eye movement or EMG artifact with amplitude exceeding 75 microvolts (thus excluding the epochs containing a high amplitude K-complex during N2) and single epochs recorded during transition phases between two sleep stages were also rejected from the analyzes. Trials were rejected if the sentence was not fully presented during the vigilance state of interest. The resulting averaged number of trials per experimental condition per participant are given in the Supplementary Information.

Then, ERPs were averaged across participants to create grand averaged ERPs (used for illustrative purposes).

Statistical calculations were performed on averaged traces from each individual on the mean amplitudes (from baseline) within time-windows of interest estimated though preliminary analyzes using latency windows of 50 ms in the 0–1400 ms range [40]. To this aim, repeated-measures analyzes of variance (ANOVA) were used for statistical assessment. To test the cortical distribution of the effects, six regions of interest (ROIs) were selected as levels of a topographic within-participants factor: left (F3, F7) and right (F4, F8) frontal, left (C3, T7) and right (C4, T8) central, and left (P3, P7) and right (P4, P8) parietal. Therefore, we performed the repeated-measures ANOVAs with the following factors: Sentence Type (congruent sentence vs. short SSL incongruent sentence vs. long SSL incongruent sentence), DL (DL0 vs. DL1 vs. DL3), Vigilance State (wake, N2, R), Antero-posterior (anterior vs. central vs. posterior), and Hemisphere (left vs. right).

Note that ANOVAs including the midline electrodes were also performed. However, because no major differences were found between these two types of analyzes, we only reported those including the six ROIs. All reported *p-*values were adjusted with the Greenhouse–Geisser correction for non-sphericity, when appropriate. Scheffé tests were used for post hoc comparisons. The reported partial eta squared (η*_p_* ^2^) is a measure of effect size for ANOVAs [41], [42]. The statistical analyzes were conducted with Cleave (January 30, 2005 Version). Cleave performed automatically all Scheffé posthoc tests (corrected for multiple comparisons) on the significant main effects and on all significant interactions.

## Results

**Figure 1:**
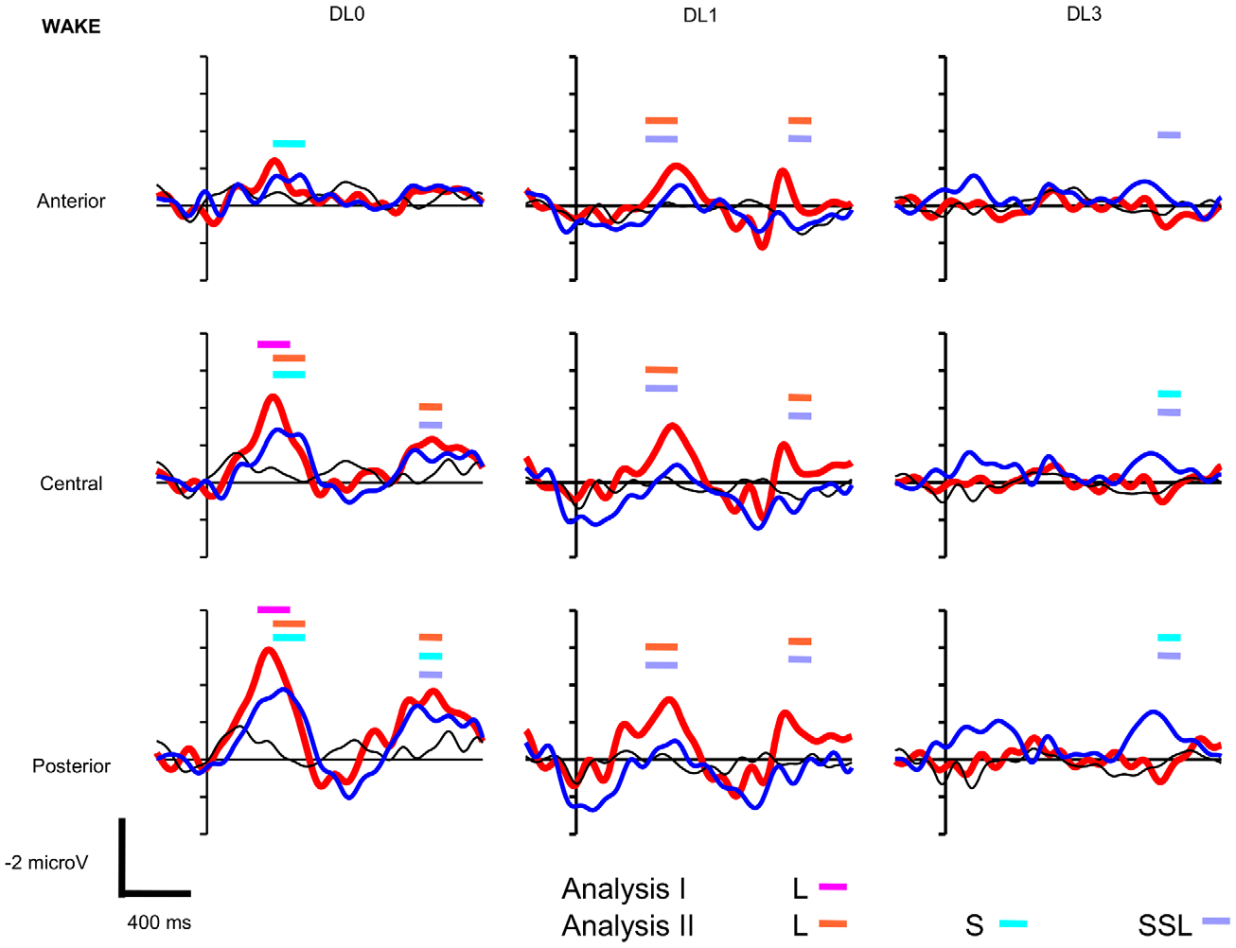
Wake grand averaged event-related potentials to long and short sentential semantic length (SSL, see Methods) (thick red and thin blue lines, respectively) and to congruent sentences (thin black lines) during wake at anterior (F3, F4, F7, F8), central (C3, C4, T7, T8), and posterior sites of the scalp (P3, P4, P7, P8) using non degraded (DL0), mildly degraded (DL1), and highly degraded (DL3) auditory sentences (N = 15 participants, vertical unit: microvolts with negativity upward, horizontal unit: milliseconds). Thick horizontal bars indicate the latencies of significant ERP effects according to Analysis I and II: ERP to **L**ong SSL incongruent sentences minus ERP to congruent sentences (**L**) significant according to Analysis I (pink) and to Analysis II (orange); ERP to **S**hort SSL incongruent sentences minus ERP to congruent sentences (**S**) significant according to Analysis II (light blue); ERP to long SSL incongruent sentences minus ERP to short SSL incongruent sentences (**SSL**) significant according to Analysis II (light purple).

**Figure 2:**
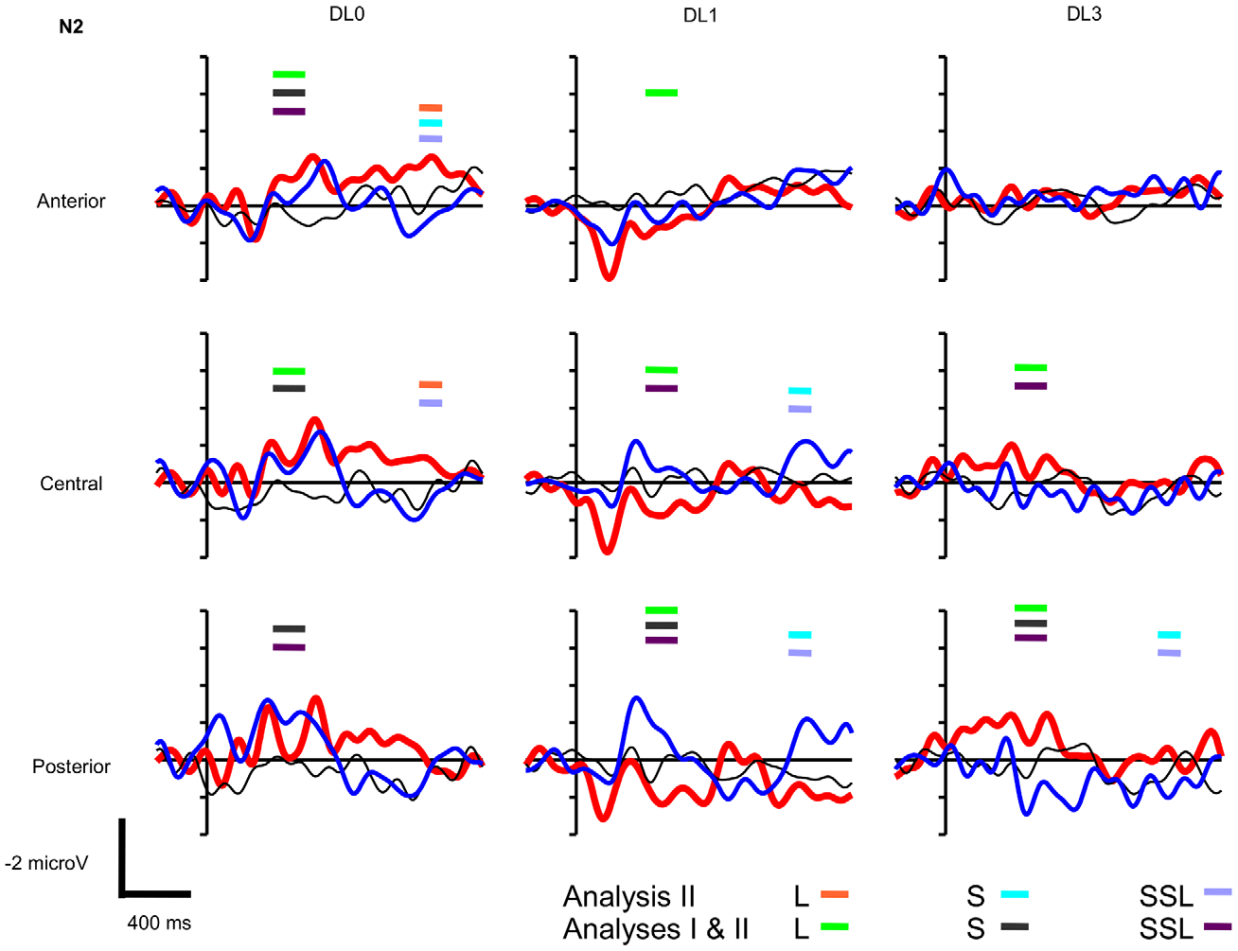
N2 grand averaged event-related potentials to long and short sentential semantic length (SSL, see Methods) (thick red and thin blue lines, respectively) and to congruent sentences (thin black lines) during N2 at anterior (F3, F4, F7, F8), central (C3, C4, T7, T8), and posterior sites of the scalp (P3, P4, P7, P8) using non degraded (DL0), mildly degraded (DL1), and highly degraded (DL3) auditory sentences (N = 15 participants, vertical unit: microvolts with negativity upward, horizontal unit: milliseconds). Thick horizontal bars indicate the latencies of significant ERP effects according to Analysis I and II: ERP to **L**ong SSL incongruent sentences minus ERP to congruent sentences (**L**) significant according to Analysis II (orange) and to Analysis I & II (green); ERP to **S**hort SSL incongruent sentences minus ERP to congruent sentences (**S**) significant according to Analysis II (light blue) and to Analysis I & II (gray); ERP to long SSL incongruent sentences minus ERP to short SSL incongruent sentences (**SSL**) significant according to Analysis II (light purple) and to Analysis I & II (dark purple).

**Figure 3:**
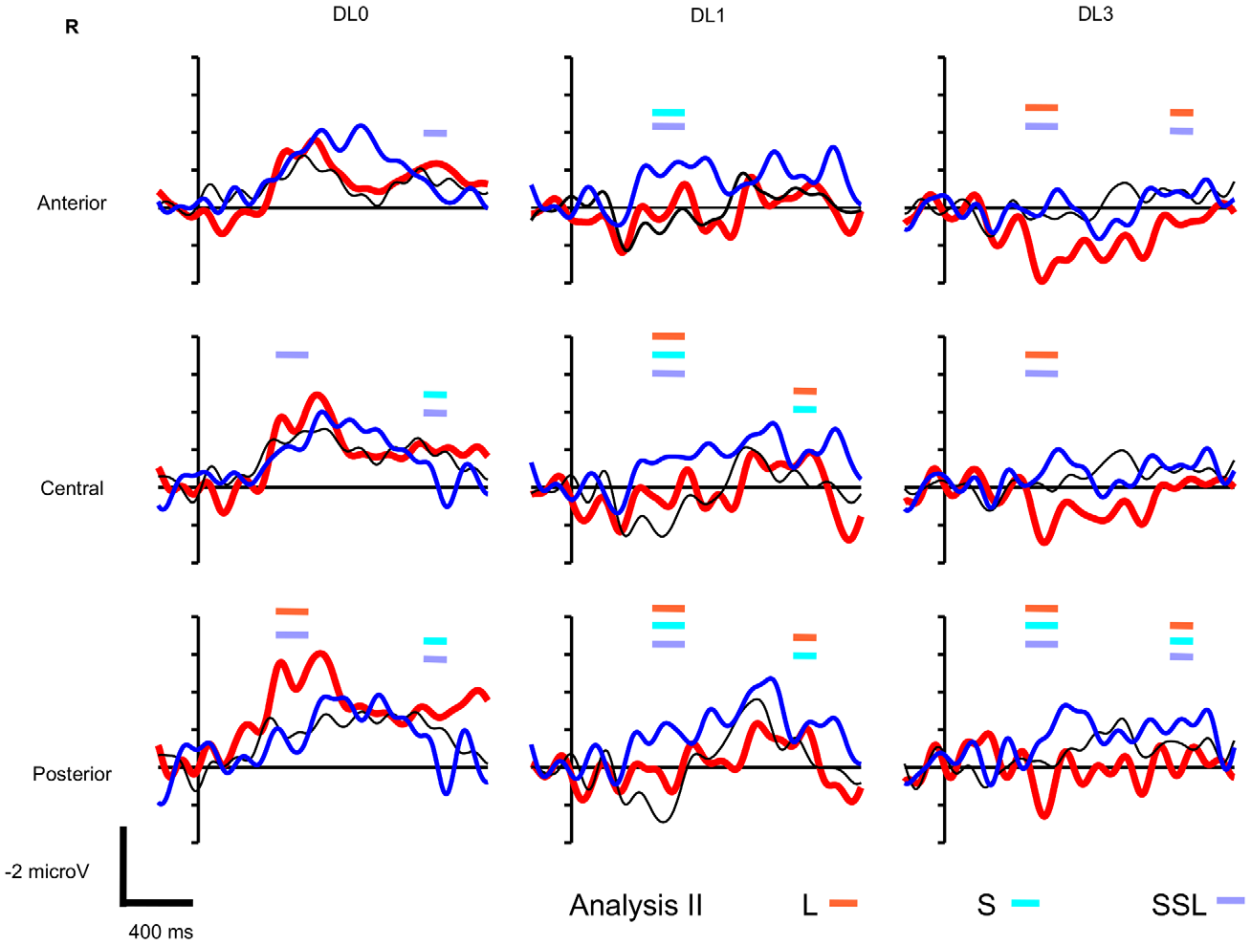
R grand averaged event-related potentials to long and short sentential semantic length (SSL, see Methods) (thick red and thin blue lines, respectively) and to congruent sentences (thin black lines) during R at anterior (F3, F4, F7, F8), central (C3, C4, T7, T8), and posterior sites of the scalp (P3, P4, P7, P8) using non degraded (DL0), mildly degraded (DL1), and highly degraded (DL3) auditory sentences (N = 15 participants, vertical unit: microvolts with negativity upward, horizontal unit: milliseconds). Thick horizontal bars indicate the latencies of significant ERP effects according to Analysis I and II: ERP to **L**ong SSL incongruent sentences minus ERP to congruent sentences (**L**) significant according to Analysis II (orange); ERP to **S**hort SSL incongruent sentences minus ERP to congruent sentences (**S**) significant according to Analysis II (light blue); ERP to long SSL incongruent sentences minus ERP to short SSL incongruent sentences (**SSL**) significant according to Analysis II (light purple).

The paradigm includes 27 conditions, i.e. 3 factors with 3 levels (Sentence Type: congruent sentence vs. short SSL incongruent sentence vs. long SSL incongruent sentence, DL: DL0 vs. DL1 vs. DL3, and Vigilance State: wake vs. N2 vs. R), to what is added the EEG topography with Hemisphere and Antero-Posterior factors, hence a rather complex statistical model. Thus, in addition to this global analysis that allow to test interactions with the Vigilance State, we present a simpler analysis with the removal of the Vigilance State that addresses each state of vigilance separately (**Analysis I**). We present first the Analysis I and then the global analysis (**Analysis II**). The grand averaged ERPs observed during wake are presented on **Figure 1**, during N2 on **Figure 2**, and during R on **Figure 3**. These figures display the time-windows were significant ERP effects have been found according to Analyzes **I** and **II**.

### Analysis I

For each state of vigilance, a repeated-measures ANOVA with Sentence Type (congruent sentence vs. short SSL incongruent sentence vs. long SSL incongruent sentence), DL (DL0 vs. DL1 vs. DL3), Antero-posterior (anterior vs. central vs. posterior), and Hemisphere (left vs. right) within-participants factors was performed.

#### Wake

The repeated-measures ANOVA did not show any effect of, or interaction with Sentence Type during the first 250 ms of the response.

Between 250 and 500 ms, there was a Sentence Type x Antero-posterior interaction [*F* (4, 56) = 8.28, *p* = .003, η*_p_* ^2^ = .372]. Post hoc comparisons (*ps*<.05) indicated the following effects (independently of the DL):

1. an N400 congruency effect (i.e. larger negativity to incongruent compared to congruent sentences at a latency of about 400 ms with a centroposterior topography) at central (−0.62 microV) and posterior sites (−0.98 microV) for long SSL incongruent sentences;
2. an N400 effect at posterior sites (−0.56 microV) for short SSL sentences.
3. non significant (*ps*>.06) SSL effect (i.e. ERP to long minus short SSL incongruent sentences).

Between 300 and 450 ms, there was a significant Sentence Type x Degradation interaction [*F* (4,56) = 5.07, *p* = .005, η*_p_* ^2^ = .266]. Post hoc comparisons (*ps*<.05) indicated an N400 effect at DL0 for long (but not short) SSL sentences (−1.45 microV), no significant (*p* = .97) SSL effect, and no significant ERP effect at DL1 and DL3 (all *ps*>.93).

Finally, between 850 and 950 ms, we found a significant Sentence Type x Antero-posterior interaction [*F* (4, 56) = 6.67, *p* = .003, η*_p_* ^2^ = .323]. Post hoc comparisons (*ps*<.05) indicated a SSL effect (0.43 microV) at posterior sites. Other ERP effects were not significant (all *ps*>.07).

All these effects are summarized in **Table 1**.

**Table 1:** Significant ERP effects according to the post hoc tests of Analysis I.

		DL independent		DL0	DL1	DL3
W		250–500 ms	850–950 ms	300–450 ms	300–450 ms	300–450 ms
	Lg	−0.80 (CP)	*n.s.*	−1.45	*n.s.*	*n.s.*
	S	−0.56 (P)	*n.s.*	*n.s.*	*n.s.*	*n.s.*
	SSL	*n.s.*	0.43 (P)	*n.s.*	*n.s.*	*n.s.*
N2				400–550 ms	400–550 ms	400–550 ms
	Lg			−0.98 (AC)	0.70	−0.88 (P)
	S			−1.08 (P)	−0.78 (P)	0.81 (P)
	SSL			0.86 (P)	1.33 (CP)	−1.20 (CP)
R		600–750 ms				
	Lg	*n.s.*				
	S	*n.s.*				
	SSL	*n.s.*				

Note: Significant ERP effects according to the post hoc tests of Analysis I (*p*<.05) in microV with their topography in parenthesis to the presentation of non degraded (DL0), mildly degraded (DL1), and highly degraded (DL3) auditory sentences during wake, N2, and R. A: Anterior sites (F3, F4, F7, F8), C: Central sites (C3, C4, T7, T8), P: Posterior sites (P3, P4, P7, P8), CP: Central and Posterior sites, AC: Anterior and Central sites, *n.s.*: non-significant ERP effect (*p*>.05), Lg: ERP (averaged in the above-mentioned time windows) to **long** SSL incongruent sentences minus ERP to congruent sentences, S: ERP to **short** SSL incongruent sentences minus ERP to congruent sentences, SSL: ERP to long minus short SSL incongruent sentences, W: Wake, DL independent: ERP effects independent from the DL (i.e. from an interaction that did not include the DL factor).

#### Sleep stage 2

The repeated-measures ANOVA did not show any effect of, or interaction with Sentence Type during the first 400 ms of the response.

Between 400 and 550 ms, there was a Sentence Type x Degradation x Antero-posterior interaction [*F* (8,112) = 4.98, *p* = .002, η*_p_* ^2^ = .262]. Post hoc comparisons (*ps*<.05) indicated the following effects:

1. at DL0: (a) a negativity effect (i.e. larger negativity to incongruent compared to congruent sentences) at anterior (−1.11 microV) and central sites (−0.86 microV) for long SSL sentences and at posterior sites (−1.08 microV) for short SSL sentences, (b) a SSL effect at posterior sites (0.86 microV);
2. at DL1: (a) a positivity effect (i.e. a larger positivity for incongruent sentences compared to congruent sentences) at all scalp sites (0.70 microV) for long SSL sentences, (b) a negativity effect at posterior sites (−0.78 microV) for short SSL sentences, (c) a SSL effect at central (1.09 microV) and posterior sites (1.56 microV);
3. at DL3: (a) a negativity effect at posterior sites (−0.88 microV) for long SSL sentences, (b) a positivity effect at posterior sites (0.81 microV) for short SSL sentences, (c) a SSL effect at central (−0.70 microV) and posterior sites (−1.70 microV).

All these effects are summarized in **Table 1**.

#### Paradoxical Sleep

The repeated-measures ANOVA did not show any effect of, or interaction with Sentence Type during the first 600 ms of the response.

Between 600 and 750 ms, there was a main effect of Sentence Type [*F* (2,28) = 5.42, *p* = .015, η*_p_* ^2^ = .279]. Post hoc comparisons did not reach significance (all *ps*>.69). None of the interactions with Sentence Type reached significance (all *ps*>.14).

### Analysis II

A repeated-measures ANOVA with Sentence Type (congruent sentence vs. short SSL incongruent sentence vs. long SSL incongruent sentence), DL (DL0 vs. DL1 vs. DL3), Vigilance State (waking, N2, R), Antero-posterior (anterior vs. central vs. posterior), and Hemisphere (left vs. right) within-participants factors did not show any effect of, or interaction with Sentence Type during the first 400 ms of the response.

Between 400 and 550 ms, there was a Sentence Type x Degradation x Vigilance State x Antero-posterior interaction [*F* (16, 224) = 2.95, *p* = .007, η*_p_* ^2^ = .174]. Post hoc comparisons (*ps*<.05) indicated the following effects:

1. during wake: (a) at DL0: an N400 effect at central (−1.16 microV) and posterior sites (−1.24 microV) for long SSL sentences and at all scalp sites (−1.04 microV) for short SSL sentences, and no significant SSL effect (*ps*>.99), (b) at DL1: an N400 effect at all scalp sites (−1.00 microV) for long SSL sentences, no effect for short SSL sentences (*ps*>.99), and a SSL effect at all scalp sites (−0.82 mivroV), (c) at DL3: no significant (*ps*>.99) ERP effect.
2. during N2: (a) at DL0: a negativity effect at anterior (−1.11 microV) and central sites (−0.86 microV) for long SSL sentences, a negativity effect at all scalp sites (−0.76 microV) for short SSL sentences, a SSL effect at anterior (−0.58 microV) and posterior sites (0.86 microV), (b) at DL1: a positivity effect at all scalp sites (0.70 microV) for long SSL sentences, a negativity effect at posterior sites (−0.78 microV) for short SSL sentences, and a SSL effect at central (1.09 microV) and posterior sites (1.56 microV), (c) at DL3: a negativity effect at central (−0.56 microV) and posterior sites (−0.88 microV) for long SSL sentences, a positivity effect at posterior sites (0.81 microV) for short SSL sentences, and a SSL effect at central (−0.70 microV) and posterior sites (−1.70 microV).
3. during R: (a) at DL0: a negativity effect at posterior sites (−1.58 microV) for long (but not for short, *ps*>.99) SSL sentences, and a SSL effect at central (−0.70 microV) and posterior sites (−1.80 microV), (b) at DL1: a negativity effect at central (−0.74 microV) and posterior sites (−0.82 mivroV) for long SSL sentences, a negativity effect at all scalp sites (−1.63 microV) for short SSL sentences, and a SSL effect at all scalp sites (0.95 microV), (c) at DL3: a positivity effect at all scalp sites (1.08 microV) for long SSL sentences, a negativity effect at posterior sites (−0.63 microV) for short SSL sentences, and a SSL effect at all scalp sites (1.56 microV).

Between 550 and 700 ms, there was a significant Sentence Type x Degradation x Antero-posterior x Hemisphere interaction [*F* (8,112) = 2.64, *p* = .022, η*_p_* ^2^ = .158]. Post hoc comparisons (*ps*<.05) indicated the following effects (independently of the State of Vigilance):

1. at DL0: (a) a negativity effect at all scalp sites except the right anterior region (−0.20 microV) for long SSL sentences, (b) a negativity effect at all scalp sites (−0.51 microV) for short SSL sentences, and (c) a SSL effect at the left anterior sites (0.22 microV);
2. at DL1: (a) a negativity effect at left hemisphere sites (−0.37 microV) for short (but not for long, *ps*>.99) SSL sentences, and (b) a SSL effect at the left hemisphere sites (0.42 microV);
3. at DL3: (a) a positivity effect at left anterior sites (0.33 microV) for long (but not for short, *ps*>.12) SSL sentences, and (b) a SSL effect at the right anterior sites (0.78 microV).

Finally, between 1100 and 1200 ms, we found a significant Sentence Type x Degradation x Vigilance State x Antero-posterior interaction [*F* (16, 224) = 2.56, *p* = .014, η*_p_* ^2^ = .155]. Post hoc comparisons (*ps*<.05) indicated the following effects:

1. during wake: (a) at DL0: a negativity effect at central (−0.83 microV) and posterior sites (−1.31 microV) for long SSL sentences, a negativity effect at posterior sites (−0.77 microV) for short SSL sentences, and a SSL effect at central (−0.48 microV) and posterior sites (−0.54 microV), (b) at DL1: a negativity effect at all scalp sites (−0.77 microV) for long (but not for short, *ps*>.99) SSL sentences, and a SSL effect at all scalp sites (−0.85 microV), (c) at DL3: a negativity effect at central (−0.60 microV) and posterior sites (−0.89 microV) for short (but not long) SSL sentences, and a SSL effect at all scalp sites (0.87 microV).
2. during N2: (a) at DL0: a negativity effect at anterior (−0.94 microV) and central (−0.69 microV) sites for long SSL sentences, a positivity effect at anterior sites (0.63 microV) for short SSL sentences, and a SSL effect at anterior (−1.57 microV) and central sites (−1.00 microV), (b) at DL1: a negativity effect at central (−1.09 microV) and posterior sites (−1.34 microV) for short (but not for long, *ps*>.99) SSL sentences, and a SSL effect at central (1.26 microV) and posterior sites (1.66 microV), (c) at DL3: a positivity effect at posterior sites (0.64 microV) for short (but not for long, *ps*>.99) SSL sentences , and a SSL effect at posterior sites (−0.93 microV).
3. during R: (a) at DL0: a positivity effect at central (0.77 microV) and posterior sites (0.93 microV) for short (but not for long, *ps*>.07) SSL sentences, and a SSL effect at all scalp sites (−0.96 microV), (b) at DL1: a negativity effect at central (−0.74 microV) and posterior sites (−0.52 microV) for long SSL sentences, a negativity effect at central (−0.71 microV) and posterior sites (−0.75 microV) for short SSL sentences, and no significant SSL effect (*ps*>.99), (c) at DL3: a positivity effect at anterior (0.73 microV) and posterior sites (0.56 microV) for long SSL sentences, a negativity effect at posterior sites (−0.50 microV) for short SSL sentences, and a SSL effect at anterior (0.70 microV) and posterior sites (1.06 microV).

All these effects are summarized in **Table 2**.

**Table 2:** Significant ERP effects according to the post hoc tests of Analysis II.

		DL0			DL1			DL3		
		400–550 ms	550–700 ms	1100–1200 ms	400–550 ms	550–700 ms	1100–1200 ms	400–550 ms	550–700 ms	1100–1200 ms
S indep.	Lg		−0.20 (*)			*n.s.*			0.33 (LA)	
	S		−0.51			−0.37 (L)			*n.s.*	
	SSL		0.22 (LA)			0.42 (L)			0.78 (RA)	
W	Lg	−1.20 (CP)		−1.07 (CP)	−1.00		−0.77	*n.s.*		*n.s.*
	S	−1.04		−0.77 (P)	*n.s.*		*n.s.*	*n.s.*		−0.75 (CP)
	SSL	*n.s.*		−0.51 (CP)	−0.82		−0.85	*n.s.*		0.87
N2	Lg	−0.98 (AC)		−0.82 (AC)	0.70		*n.s.*	−0.72 (CP)		*n.s.*
	S	−0.76		0.63 (A)	−0.78 (P)		−1.22 (CP)	0.81 (P)		0.64 (P)
	SSL	−0.58 (A) 0.86 (P)		−1.29 (AC)	1.33 (CP)		1.46 (CP)	−1.20 (CP)		−0.93 (P)
R	Lg	−1.58 (P)		*n.s.*	−0.78 (CP)		−0.63 (CP)	1.08		0.73 (A) 0.56 (P)
	S	*n.s.*		0.85 (CP)	−1.63		−0.73 (CP)	−0.63 (P)		−0.50 (P)
	SSL	−1.25 (CP)		−0.96	0.95		*n.s.*	1.56		0.70 (A) 1.06 (P)

Note: Significant ERP effects according to the post hoc tests of Analysis II (*p*<.05) in microV with their topography in parenthesis to the presentation of non degraded (DL0), mildly degraded (DL1), and highly degraded (DL3) auditory sentences during wake, N2, and R. A: Anterior sites (F3, F4, F7, F8), C: Central sites (C3, C4, T7, T8), P: Posterior sites (P3, P4, P7, P8), L: Left hemisphere sites (F3, F7, C3, T7, P3, P7), CP: Central and Posterior sites, AC: Anterior and Central sites, RA: Right anterior sites (F4, F8), LA: Left anterior sites (F3, F7),

* : all scalp sites except the right anterior region, *n.s.*: non-significant ERP effect (*p*>.05), Lg: ERP (averaged in the above-mentioned time windows) to **long** SSL incongruent sentences minus ERP to congruent sentences, S: ERP to **short** SSL incongruent sentences minus ERP to congruent sentences, SSL: ERP to long minus short SSL incongruent sentences, W: Wake, S indep.: ERP effects independent from the Vigilance State (i.e. from an interaction that did not include the Vigilance State factor).

## Discussion

The main aim of the present ERP study was to investigate if working memory (WM) for speech processing remained during sleep. Our findings were as follows:

1. We found a larger N400 component in response to sentences (without noise, i.e. DL0 condition) ending with a semantically incongruent word compared to a congruent word (N400 effect) during N2 and R. This finding replicates the results of Ibáñez et al. [14], showing that speech processing remains during sleep.
2. We showed for the first time speech-in-noise processing during N2 and R. Indeed, the N400 effect was observed during N2 and R in a mild noise condition (DL1). Furthermore, we found attenuated ERPs effects during N2 and R in a strong noise condition (DL3) that could be interpreted as a small N400 effects. These ERP effects at DL3 found during sleep but not during wake suggest that sleep and wake speech-in-noise processing seem to activate qualitatively different mechanisms.
3. Using a gating paradigm that assessed the sentential semantic length (SSL, see Methods) and coupling two parameters to increase the load on WM during speech perception (i.e. the use of a mild background noise and long SSL sentences that require more words to be stored in WM than short SSL sentences), we found that, according to the N400 effect, speech perception was impaired by a moderate background of noise (DL1) during N2 and R compared to wake. This impairment was interpreted as reflecting a reduced WM capacity for speech processing during these sleep stages compared to wake.

### Speech processing during sleep

Sleep ERP studies testing semantic processing have mainly used single word or word pair paradigms (for a review, see [1]). To our knowledge, the report of Ibáñez et al. [14] is the only one that demonstrates semantic processing of sentences during sleep. Using sentences ending on a semantically congruent or incongruent word, they reported an N400 effect during N2 and R (and during wake, as control data). Here we report a similar result. At wake our material of semantically incongruent and congruent sentences elicited the expected centro-parietal N400 effect [12]. During N2 and R, we found an N400 effect between 400 ms and 700 ms, that is, at a delayed time-window compared to wake. This is in agreement with the study of Ibáñez et al. [14] who also reported N400 effects during sleep between 400 ms and 700 ms. Unlike Ibáñez et al. [14] who found a left frontal N400 effect during N2 and R, we report a centro-parietal N400 effect, i.e. a topography closer to the one expected at wake. The N400 research has reported that the topography of this component, although often reported at centro-parietal sites, may vary, possibly because of its polymodal context-dependent characteristics, being elicited by several cognitive processes with multiple generating sources [43], [44]. The sentences of Ibáñez et al. [14] were sentential definitions (e.g. “It has a tail and lives in the water, it is a gull”.), thus were not sentences from the usual spoken language (as our sentences). Ibáñez's sentences ended on a word defined by the sentential context (congruent sentences) or by a word unrelated to the sentential definition (incongruent sentences). This specific material may have resulted in a different semantic processing (hence possibly, a different topographic activation) compared to the one elicited during usual spoken sentence processing and hence be responsible for the left frontal distribution. The fact that Ibáñez et al. [14] found a left frontal topography not only during sleep but also during wake suggests that the topographical difference between their study and ours depends more on the used material than on the vigilance state.

### Speech-in-noise perception during sleep

Within an ecological environment, speech is usually perceived within noise. Although ERP sleep researchers recommend exploring sleep cognition with more ecologically valid designs [45], to date, speech-in-noise perception has never been tested during sleep. In the present study, the effect of noise on speech perception was assessed using three experimental conditions: sentences presented without noise (sentence degradation level 0: DL0), with mild noise (degradation level 1: DL1), and with strong noise (degradation level 3: DL3) (see Methods). Speech perception during wake in these three conditions has been previously tested [46], indicating delayed N400 effects at DL1 compared to DL0 and a lack of N400 effect at DL3. Our results during wake closely replicated these findings, with a delayed N400 effect at DL1 compared to DL0 and the lack of N400 effect at DL3. The novelty of the present study was to investigate the perception of these noisy sentences (and to assess WM) during sleep.

During sleep, we showed that, similarly to wake, the N400 effect depends on the level of noise. While, with mildly degraded sentences (DL1, allowing still easy comprehension at wake, see [46]) a clear N400 effect could be recorded during N2 (with short SSL sentences) and during R (with long and short SSL sentences), the use of a stronger noise (DL3) attenuated the ERP effects. Responses at DL3 showed nevertheless significantly larger negativities to incongruent compared to congruent sentences within the 400 ms to 700 ms range at centro-posterior sites during N2 and R that could be interpreted as small N400 effects. This result was rather unexpected. Indeed, one would guess that if a semantic stimulus is already hard to perceive during wake (see the behavioral data at DL3 in [46]) and does not elicit clear ERP variation according to its contextual semantic congruency, it is unlikely to find ERP congruency effects during N2 and R. At DL3, the behavioral data of Daltrozzo et al. [46] indicated that, during wake, speech was hard to be perceive but was nevertheless processed through automatic mechanisms. The lack of an N400 effect at wake together with an N400 effect during sleep (N2, R) suggest that sleep and wake cognition may differ qualitatively. In particular, while wake mechanisms of speech processing activated at DL3 (i.e. robust to noise) are not generators of the N400 (speech processing being observed only through behavioral data, [46]), sleep mechanisms of speech processing activated at DL3 (i.e. also robust to noise) would be qualitatively different, as reflected by their ability to modulate the N400 (further discussion in the next section).

### Working memory for speech perception during sleep

The literature suggests that cortical areas recruited during WM tasks, such as the dorsolateral prefrontal cortex, are less activated during sleep than during wake [17]–[19]. According to Baddeley [2], [3], WM would be activated during speech perception, via a “central executive system” and a slave system referred to as the “phonological loop”. Therefore, speech perception can be used to explore WM in N2 and R.

With this aim, WM load was manipulated with two experimental parameters: the SSL and sentential degradation. Our incongruent sentences were divided into two groups: short and long SSL incongruent sentences. In addition, sentences were presented at three degradation levels (DL0, DL1, and DL3). A higher load on WM was required with long compared to short SSL sentences and with increasing sentential degradation. Thus, we were able to compare the N400 responses in response to sentences according to the load on WM.

#### Sentences without degradation (DL0)

When sentences were perceived without noise (DL0), wake data showed a larger N400 effect to the long SSL incongruent sentences compared to the short SSL incongruent sentences. This difference suggests that long SSL sentences were more incongruent than short SSL sentences. However, our first pilot behavioral study (see Supplementary Information) suggests that the short SSL sentences were more incongruent than the long. Taking our N400 and behavioral data together, the difference of N400 between short and long SSL sentences would rather reflect a WM load difference between short and long SSL sentences rather than a difference of semantic congruency. This N400 difference at DL0 during wake (i.e. the control data) is not negligible and has to be taken into account for the interpretation of our data in all the other experimental (test) conditions.

During N2 and R, a N400 effect was also found with long and short SSL sentences. As expected from Ibáñez et al. [14] (see discussion below), these results indicate that the increased WM load to process long compared to short SSL sentences did not saturate the WM capacity in these sleep stages.

#### Sentences with mild degradation (DL1)

During wake, data at DL1 showed a similar pattern as data at DL0, with a larger N400 effect to long compared to short SSL sentences. Unlike wake data, N2 and R data showed a different pattern between DL1 and DL0.

During N2, only the short SSL sentences showed an N400 effect, while long SSL sentences showed a larger positivity to incongruent compared to congruent sentences that might reflect a failure to fully process the sentences because of a too limited WM capacity. Indeed, the cognitive processing of the speech material may be impaired/disrupted, with for instance, an incomplete syntactic analysis of the sentential context. If this syntactic analysis is not completed, the final word of the sentence may not be perceived as a syntactically congruent word. During wake, when a word is perceived within a syntactically incongruent sentential context, a P600 is observed (for a review, see [47]). Thus, provided that similar mechanisms of syntactic processing are activated at the presentation of sentences during sleep, the larger positivity to long SSL incongruent sentences compared to congruent sentences observed during N2 at DL1 would reflect a syntactic mismatch due to incomplete sentential processing (including incomplete syntactic analysis) as the WM load reaches saturation.

During R, both long and short SSL sentences showed an N400 effect. However, short SSL sentences elicited a larger N400 effect than long SSL sentences, while the opposite tendency (larger effect for long compared to short SSL sentences) was found at DL0 (**Figure 3**).

These sleep (N2 and R) data differences between DL0 and DL1 would arise from a combined effect: the reduced WM capacity during sleep compared to wake together with the increased load on WM required to process degraded sentences. Indeed, speech-in-noise perception requires more WM capacity than speech perception without noise [23]–[28]. Thus, the WM capacity during sleep would be sufficient for the perception of non-degraded sentences (DL0), but would be saturated or close to saturation during N2 and R when sentences are presented within mild noise (DL1). In contrast, during wake, the N400 effect is not reduced between DL0 and DL1, neither for short nor for long SSL sentences, suggesting that saturation of the WM capacity was not reached during wake perception of speech-in-noise at DL1. If at DL1, WM capacity reached (or was close to) saturation during sleep but not during wake, it is likely that the WM capacity was smaller during sleep than during wake.

Although the study of Ibáñez et al. [14] did not aim to assess WM (leading to suboptimal control of WM load), their data are nevertheless in agreement ours as concerns WM during sleep. Ibáñez et al. [14] recorded the N400 to four types of sentences: congruent sentences and 3 types of incongruent sentences where the final word was either incongruent with the beginning, the end, or the full sentence. Ibáñez et al. [14] found a similar N400 effect during sleep (N2 and R) independently of the type of incongruent sentences. It is likely that the sentences where the target was incongruent with the beginning of the sentence required more words to be stored in WM for the perception of the semantic incongruence than sentences where the target was incongruent with the end of the sentence. The lack of a significant difference of N400 effect between these two experimental conditions during N2 and R suggests that the WM capacity, if reduced during N2 and R, was not small enough to impair the perception of the semantic incongruence of these sentences. This interpretation fits well with our result. Indeed, as mentioned above, our data support the conclusion that the WM capacity during N2 and R allows the processing of short and long SSL sentences when they are presented without noise (DL0), that is, the WM capacity for speech processing did not reach or approach saturation. Saturation was reached or approached only if our sentences were presented within noise, a condition that was not explored by Ibáñez et al. [14].

#### Sentences with strong degradation (DL3)

During wake, our data replicated Daltrozzo et al. [46] showing a lack of N400 effect. During N2 and R, we found responses that could be interpreted as small N400 effects, suggesting a remaining but qualitatively different speech processing compared to wake. Indeed, differences in qualitative speech cognition during sleep compared to wake have already been reported [15], [48], [49]. Our data suggest that the impaired speech processing observed at DL1 would be compensated by other speech processing mechanisms when the noise is further increased (DL3). In contrast to wake [46], sleep mechanisms of speech processing activated at DL3 would be able to modulate the N400. This difference further suggests that (noise robust) speech processing mechanisms of sleep would differ qualitatively from (noise robust) speech processing mechanisms of wake.

Additionally, as the presumed N400 effects at DL3 during sleep were found only with long SSL sentences during N2 and only with short SSL sentences during R, qualitative differences of noise robust speech cognition between N2 and R may also exist, a conclusion in agreement with previous studies [15], [48], [49].

Provided that mechanisms of syntactic processing similar to the wake can be activated by the presentation of sentences during sleep, the larger positivity to long SSL incongruent sentences compared to congruent sentences found during R might reflect a P600 to a syntactic mismatch. Indeed, as mentioned above (see Discussion, previous section), a failure to fully process the sentences because of a too limited WM capacity may include an incomplete syntactic processing of the sentence. In such case, the final word of the sentence may not be perceived as a syntactically congruent word.

During N2, the larger positivity to short SSL incongruent sentences compared to congruent sentences may also reflect a P600 to syntactic mismatch, and hence, a limited WM capacity. The presumed N400 effect to long SSL incongruent sentences may be elicited by uncertainties arising from the content of the sentential context (due to the strong noise), which mobilize neuronal assemblies to scan for further information needed to build a “model of possible content” [50], p59.

#### Specificity of the SSL and the sentence degradation parameters for the WM load manipulation

The SSL manipulation was designed on the basis of a gating paradigm (see Supplementary Information) with the aim to modulate the WM load. We checked the specificity of this parameter for WM load.

The contextual constraint (i.e. the degree to which the context establishes an expectation for a particular upcoming word) is known to modulate the N400 and is generally estimated empirically by the cloze probability [12]. Since the cloze probability did not differ significantly between short and long SSL incongruent sentences (see Methods), the contextual constraint was not confounded with the SSL manipulation.

The number of words of the sentential context is obviously not independent from the SSL, i.e. the minimum number of words of the sentential context required to perceive a sentence as semantically incongruent: short SSL incongruent sentences had a context containing fewer words than long SSL incongruent sentences (see Methods). Therefore, the SSL manipulation was confounded with the number of words of the sentential contexts. Similarly, the sentence duration was confounded with the SSL parameter. Short SSL sentences had a shorter duration than long SSL sentences (see Methods).

In summary, we believe that: (i) contextual constraint and SSL were independent parameters. (ii) the SSL had two covariates: the number of words of the sentential contexts and the sentence duration.

In addition to the SSL, the load on WM was manipulated with sentential degradation. Each sentence being presented in the three degradation conditions (i.e. DL0, DL1, and DL3) across participants, the variation of load on WM across levels of degradation could not be confounded with contextual constraint, number of words of the sentential contexts, or sentence duration.

To the authors' knowledge, the sound level cannot modulate the load on WM, and hence be a confounding parameter. Furthermore, the sound level did not vary between levels of SSL or levels of degradation, all sentences being presented at a normalized sound level (across our full material of sentences, see Methods).

#### Conclusion

Our study investigated for the first time the WM during sleep by manipulating its load through the SSL and through sentential degradation. We showed that: (1) sentential processing remains during sleep (replicating Ibáñez et al. [14]) even when speech is perceived within a background of noise (i.e. a more ecologically valid experimental condition), (2) speech-in-noise cognition would involve qualitatively different noise-robust mechanisms between wake, N2, and R, and (3) WM is preserved but with a smaller capacity during sleep (N2 and R) compared to wake.

Further explorations of sleep-specific mechanisms could be pursued via neuroimaging studies by investigating how the dorsolateral prefrontal and parietal cortices are activated during sentential processing when the load on WM is strongly increased.

## Supporting Information

Methods S1 **Pilot studies.**
(DOC)

Table S1 **Averaged number of trials per experimental condition per participant.**
(DOC)

Figure S1 **Polysomnographic samples during wake (upper panel), sleep stage 2 (middle panel), and paradoxical sleep (lower panel).** Electroencephalographic (EEG) signals from 18 Ag-AgCl electrodes (International 10–20 system sites: Fz, Cz, Pz, Oz, F7, F8, F3, F4, C3, C4, T7, T8, P7, P8, P3, P4, O1, O2) referenced to the nose, VE: Vertical Electrooculogram, HE: Horizontal Electrooculogram, EM: Electromyogram (N = 15 participants, vertical unit: microvolts with positivity upward, horizontal unit: second).
(TIF)

Figure S2 EEG spectra during wake (dotted line), sleep stage 2 (black solid line), and paradoxical sleep (gray solid line) (N = 15 participants, vertical unit: 10*log_10_(microvolts^2^/Hz), horizontal unit: Hz).
(TIF)
