# Supplementary material for: Working Memory Is Partially Preserved during Sleep
Source: PLoS One. 2012 Dec 7;7(12):e50997. doi: 10.1371/journal.pone.0050997 (PMC3517624; doi:10.1371/journal.pone.0050997)
Supplement: Methods S1 — Pilot studies. (DOC) [file pone.0050997.s001.doc]

**MATERIAL AND METHODS**

**Stimuli**

*Semantic congruency*

The semantic congruency and incongruence of the sentences were tested with a **first pilot study** that included 20 French native right-handed volunteers (17 females, *M ± SEM* = 22.5 ± 0.6 years, none of them being included in the second pilot experiment or the main experiment, see below). They were presented each of these sentences and asked to provide a score of semantic relatedness between 1 (no semantic relation between the context and the target) and 9 (context and target are highly semantically related). Seventy incongruent sentences with a score higher than 5 were excluded. The remaining 1100 incongruent sentences had a score of 1.76 ± 0.03 and the congruent sentences a score of 8.96 ± 0.01 [*t* (1149) = -51.9, *p* < .01].

*Sentential semantic length (SSL)*

A **second pilot study** was run to estimate the minimum load of sentential context that is required to be stored in WM in order to perceive each of the 1100 incongruent sentences as semantically incongruent. Thirty native French speaking, right-handed, volunteers (25 females, *M ± SEM =* 23.3 ± 1.2 years, who had not participated in the first pilot test or the main experiment) were presented a subset of all incongruent sentences (6 groups of 5 participants, one sixth of the 1100 incongruent sentences presented to each group) within a gating paradigm [1,2]. All sentences were pseudo-randomly presented per block (with different randomizations for each block and participant): the first block presented sentence endings composed of only the last three words of the sentences, the second block presented sentence endings composed of the last four words, and so on in the following blocks with a maximum of 10 blocks corresponding to the longest sentence (for instance, for the congruent sentence “Après enquête, le commissaire a arrêté le **coupable**”, the truncated presented incongruent sentences could be: “arrêté le **genou**” in the first block, “a arrêté le **cheveux**” in the second block, and “commissaire a arrêté le **diplôme**” in the third block). Participants were asked to listen carefully to each sentence ending and to guess whether this sentence ending would belong to a congruent or incongruent sentence. If participants felt that this ending belonged to a congruent sentence, they were told to provide a score of 4. If the sentence ending was perceived as belonging to a highly incongruent sentence, they were asked to give a score of 1. The score of 2 and 3 were thus intermediate judgments. If, for a given sentence, participants rated the corresponding sentence endings with a score of 1 or 2 (thus as incongruent endings) in 3 successive blocks, the test ended on-line for this incongruent sentence. The critical block used to measure the minimum number of words of the sentential context (i.e., the sentence truncated by its last word) that was required to perceive a sentence as semantically incongruent, was assumed to be the first of these three blocks (the critical block number n corresponded to the minimum number of words n+1). This estimation is referred to as "sentential semantic length" (SSL).

**Procedure**

*Data analysis*

Polysomnographic samples and EEG spectra during each vigilance state are presented in the **Figures S1** and **S2**. The averaged number of trials per experimental condition are given in **Table S1.**

**REFERENCES**

1. Grosjean F (1980) Spoken word recognition processes and the gating paradigm. Perception and Psychophysics 28(4):267-83.
2. Grosjean F (1996) Gating. Language and Cognitive Processes 11(6):597-604.
3. Kutas M; Van Petten C (1988) Event-Related Brain Potential Studies of Language. In: Ackles PK, Jennings RJ, Coles MGH, editors. .Advances in Psychophysiology. Volume 3. Greenwich, Connecticus: JAI Press Inc., p 139-187.

| **APPENDIX** | |
| --- | --- |
| **Congruent Sentences** | **Cloze Probability** |
| Brigitte a tapé son frère avec un bâton.  [Brigitte has thumbed his brother with a stick.] | 23.3 |
| A Noël, les enfants reçoivent des cadeaux.  [At Christmas, children receive gifts.] | 77.8 |
| Tous les matins, pour son petit-déjeuner, Jacques prend du café.  [Every morning for his breakfast, Jacques takes coffee.] | 23.3 |
| Souvent, après le repas, nous buvons un café.  [Often, after dinner, we drink coffee.] | 23.3 |
| On donne souvent aux lapins des carottes.  [Often rabbits are given carrots.] | 64.4 |
| En entrant, Christine a enlevé ses chaussures.  [By entering, Christine removed her shoes.] | 32.2 |
| A la récréation, Pierre lui a tiré les cheveux.  [At recess, Pierre pulled her hair.] | 37.8 |
| En sortant de la piscine, n'oublie pas de te sécher les cheveux.  [Leaving the pool, do not forget to dry your hair.] | 83.3 |
| Pendant le match, Philippe s'est foulé la cheville.  [During the match, Philippe sprained his ankle.] | 80.0 |
| L'étagère est couverte de poussière, nettoie-la avec un chiffon.  [The shelf is dusty, clean it with a dust cloth.] | 70.0 |
| Comme ses chaussures étaient sales, Martin les a nettoyées avec un chiffon.  [As his shoes were dirty, Martin cleaned them with a dust cloth.] | 24.4 |
| Souvent, après le repas, mon père fume un cigare.  [Often, after dinner, my father smokes a cigar.] | 36.7 |
| Mon frère a acheté des billets pour le concert.  [My brother bought tickets for the concert.] | 42.2 |
| Après enquête, le commissaire a arrêté le coupable.  [After investigation, the Commissioner has arrested the culprit.] | 30.0 |
| Mon père coupe le poulet avec un couteau.  [My father cut the chicken with a knife.] | 56.7 |
| Si tu as mal aux dents, tu ferais mieux d'appeler le dentiste.  [If you have a toothache, you better call the dentist.] | 80.0 |
| Au lieu de regarder la télévision, tu devrais aller faire tes devoirs.  [Instead of watching TV, you should go do your homework.] | 54.4 |
| Avant d'aller jouer, je dois terminer mes devoirs.  [Before playing, I have to finish my homework.] | 72.2 |
| Après de nombreuses tentatives, Antoine a obtenu son diplôme.  [After numerous attempts, Antoine obtained his diploma.] | 30.0 |
| Il est interdit de se pencher par la fenêtre.  [It is forbidden to lean out of the window.] | 93.3 |
| Parce qu'il a désobéi, sa mère lui a donné une fessée.  [Because he disobeyed, his mother gave him a spanking.] | 25.6 |
| Paul est tombé pendant la récréation et il s'est fait mal au genou.  [Paul fell during recess and he hurted his knee.] | 66.7 |
| Tous les jours, Paul lit le journal.  [Every day, Paul is reading the newspaper.] | 43.3 |
| Dans la forêt, le chasseur a tué un lapin.  [In the forest, the hunter killed a rabbit.] | 21.1 |
| En te couchant, pense à éteindre la lumière.  [In going to bed, think of turning off the light.] | 75.6 |
| Hier matin, des voleurs ont cambriolé la maison.  [Yesterday morning, thieves broke into the house.] | 26.7 |
| Si tu vas jouer dehors, n'oublie pas ton manteau.  [If you go playing outside, do not forget your coat.] | 21.1 |
| Cela fait trois ans qu'Irène a quitté son mari.  [It has been three years that Irene has left her husband.] | 44.4 |
| Souvent, les personnes âgées perdent la mémoire.  [Often, the elderly lose memory.] | 36.7 |
| Le marchand lui a rendu la monnaie.  [The merchant returned the money.] | 60.0 |
| En tombant du cendrier, la cigarette a brûlé la moquette.  [Falling from the ashtray, the cigarette burned the carpet.] | 43.3 |
| Avec l'école, nous avons visité un musée.  [With the school, we visited a museum.] | 41.1 |
| Après leur attaque, les pirates ont coulé le navire.  [After their attack, the pirates sank the ship.] | 42.2 |
| Prends tes ciseaux pour couper le papier.  [Take your scissors to cut the paper.] | 23.3 |
| Les déménageurs ont monté six étages à pieds en portant un piano.  [The movers mounted a six-story walk carrying a piano.] | 27.8 |
| En voyant les voleurs, ils ont appelé la police.  [Upon seeing the thieves, they called the police.] | 74.4 |
| En voyant l'incendie ils ont appelé les pompiers.  [Seeing the fire they called the fire department.] | 86.7 |
| En voyant l'accident, ils ont appelé les pompiers.  [Seeing the accident, they called the fire department.] | 31.1 |
| Dans la cuisine, Norbert prépare le repas.  [In the kitchen, Norbert makes the dinner.] | 33.3 |
| Dans la cuisine, Colette prépare le repas.  [In the kitchen, Colette makes the dinner.] | 33.3 |
| Après cinq jours de marche, ils ont atteint le sommet.  [After five days of walk, they reached the summit.] | 62.2 |
| Vous ne pouvez pas écrire sans stylo.  [You can not write without a pen.] | 30.0 |
| La maîtresse a recopié l'exercice sur le tableau.  [The teacher copied the exercise on the blackboard.] | 74.4 |
| C'est dans ce coffre que les pirates ont caché le trésor.  [It is in this coffer that the pirates have hidden the treasure.] | 51.1 |
| Ils ont visité la France pendant les vacances.  [They visited France during the holidays.] | 28.9 |
| Norbert l'a aidée à laver la vaisselle.  [Norbert helped her wash the dishes.] | 25.6 |
| Le fils de notre voisin a volé une voiture.  [Our neighbor's son stole a car.] | 24.4 |
| Si samedi il fait beau, nous pourrons laver la voiture.  [Saturday if the weather is nice, we can wash the car.] | 64.4 |
| Le vent a fait claquer violemment les volets.  [The wind slammed violently the shutters.] | 66.7 |
| La nuit, il vaut mieux fermer les volets.  [At night it is better to close the shutters.] | 51.1 |

Note: The sentences end with a semantically congruent target word. The cloze probability of the target is a percentage of participants who spontaneously complete the sentence with this target word [3]. The sentences were selected from the corpus of an unpublished thesis of phonetics (Monpiou, S. Approche du rôle du contexte phrastique dans la reconnaissance lexicale auditive des mots, Doctoral Thesis, Marc Bloch University: Strasbourg, France, 1998), wherein the cloze probability was estimated from the responses of 200 participants.
