# Supplementary material for: Working Memory Is Partially Preserved during Sleep
Source: PLoS One. 2012 Dec 7;7(12):e50997. doi: 10.1371/journal.pone.0050997 (PMC3517624; doi:10.1371/journal.pone.0050997)
Supplement: Table S1 — Averaged number of trials per experimental condition per participant. (DOC) [file pone.0050997.s002.doc]

**Table S1 :** Averaged number of trials per experimental condition per participant.

|  |  | DL0 | DL1 | DL3 | mean |
| --- | --- | --- | --- | --- | --- |
| W | L | 45 | 47 | 47 | 46 |
|  | S | 40 | 38 | 39 | 39 |
|  | C | 88 | 85 | 85 | 86 |
|  | mean | 58 | 57 | 57 | 57 |
| N2 | L | 36 | 41 | 41 | 39 |
|  | S | 32 | 37 | 34 | 35 |
|  | C | 65 | 75 | 80 | 73 |
|  | mean | 44 | 51 | 52 | 49 |
| R | L | 36 | 36 | 32 | 35 |
|  | S | 35 | 37 | 35 | 36 |
|  | C | 72 | 70 | 57 | 66 |
|  | mean | 48 | 47 | 41 | 45 |

Note : DL0: non degraded auditory sentences, DL1: mildly degraded sentences, DL3: highly degraded sentences, W: wake, N2: sleep stage 2, R: paradoxical sleep, L : long SSL incongruent sentences, S : short SSL incongruent sentences, C: congruent sentences.
