# Supplementary figures and images for: Working Memory Is Partially Preserved during Sleep
Source: PLoS One. 2012 Dec 7;7(12):e50997. doi: 10.1371/journal.pone.0050997 (PMC3517624; doi:10.1371/journal.pone.0050997)

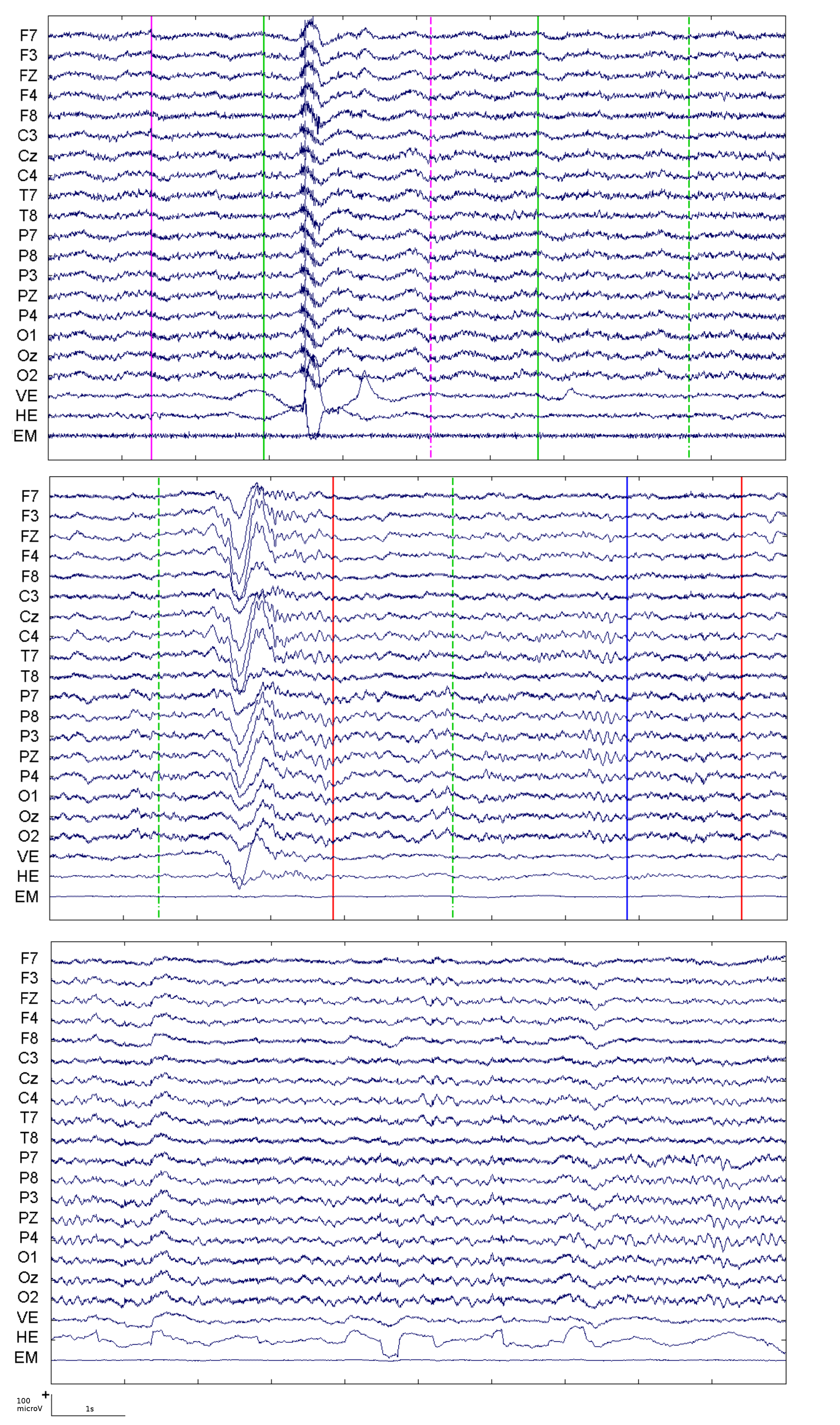

Supplement: Figure S1 — Polysomnographic samples during wake (upper panel), sleep stage 2 (middle panel), and paradoxical sleep (lower panel). Electroencephalographic (EEG) signals from 18 Ag-AgCl electrodes (International 10–20 system sites: Fz, Cz, Pz, Oz, F7, F8, F3, F4, C3, C4, T7, T8, P7, P8, P3, P4, O1, O2) referenced to the nose, VE: Vertical Electrooculogram, HE: Horizontal Electrooculogram, EM: Electromyogram (N = 15 participants, vertical unit: microvolts with positivity upward, horizontal unit: second). (TIF) [file pone.0050997.s003.tif]

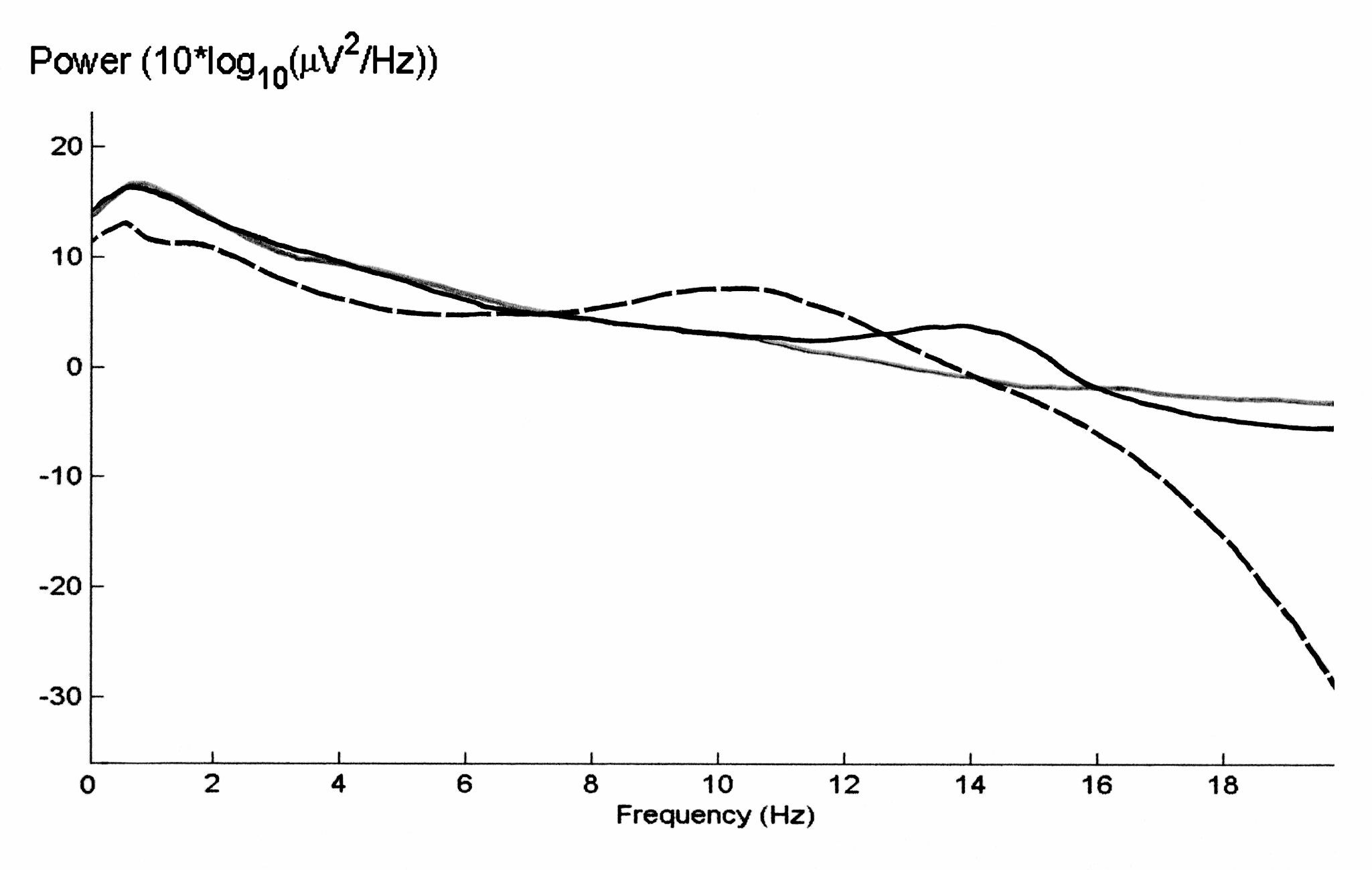

Supplement: Figure S2 — EEG spectra during wake (dotted line), sleep stage 2 (black solid line), and paradoxical sleep (gray solid line) (N = 15 participants, vertical unit: 10*log10(microvolts2/Hz), horizontal unit: Hz). (TIF) [file pone.0050997.s004.tif]
